# Supplementary material for: Structural basis of supercoiling-induced CRISPR–Cas9 off-target activity
Source: Nature. 2026 Mar 25;653(8114):627–35. doi: 10.1038/s41586-026-10255-7 (PMC13171457; doi:10.1038/s41586-026-10255-7)
Supplement: Supplementary file 1 — Supplementary Figure 1 and Supplementary Table 1 [file 41586_2026_10255_MOESM1_ESM.docx]

**Supplementary Figure 1.** Uncropped gels for all representative gels used in main and Extended Data Figures.

**Figure 1a:**

**
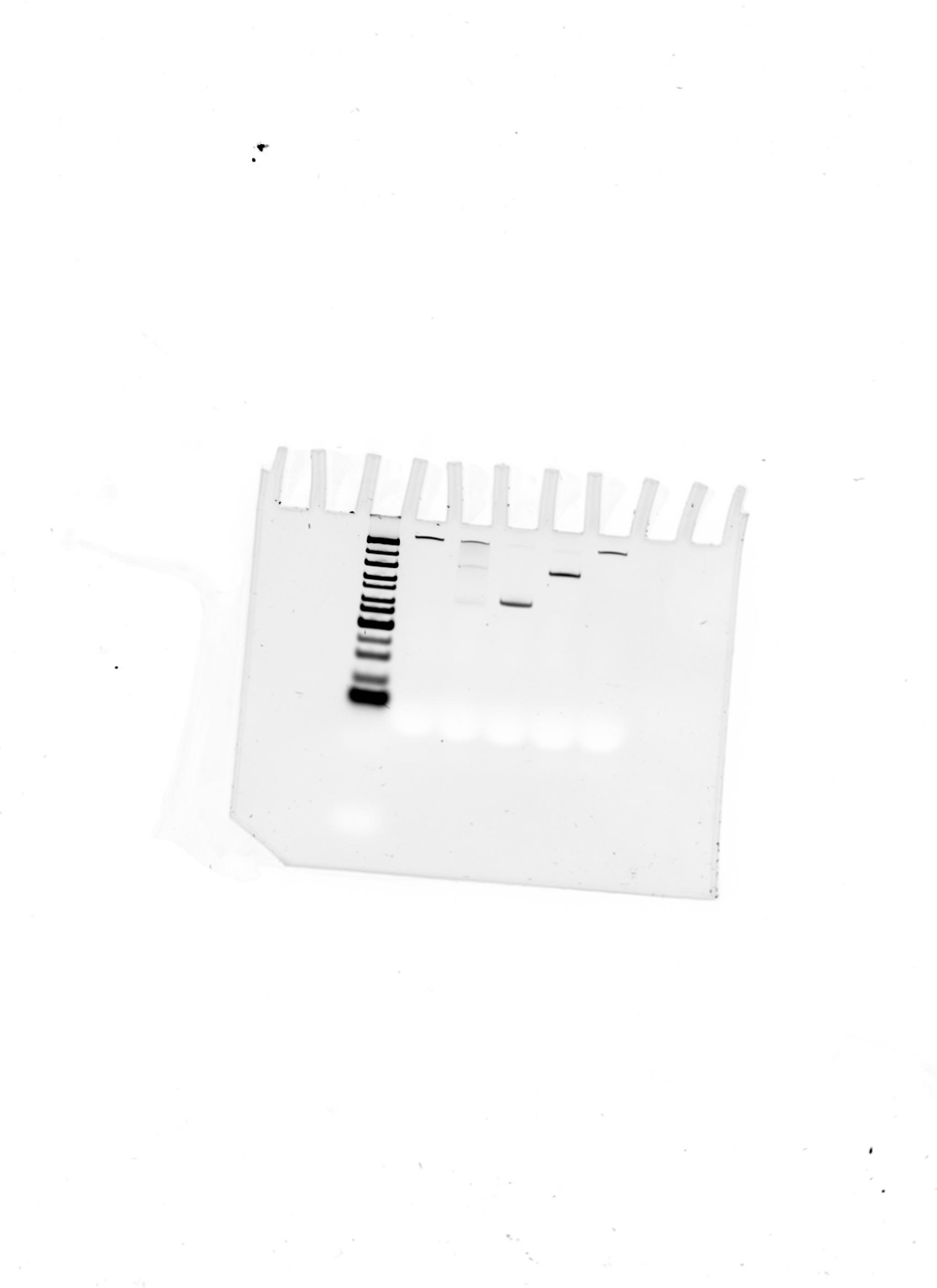
**

**Figure 1b:**


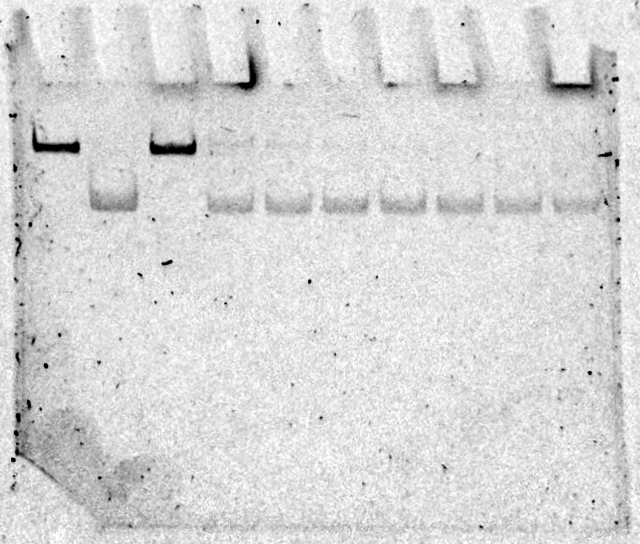


**Extended Data Figure 1b:**

**
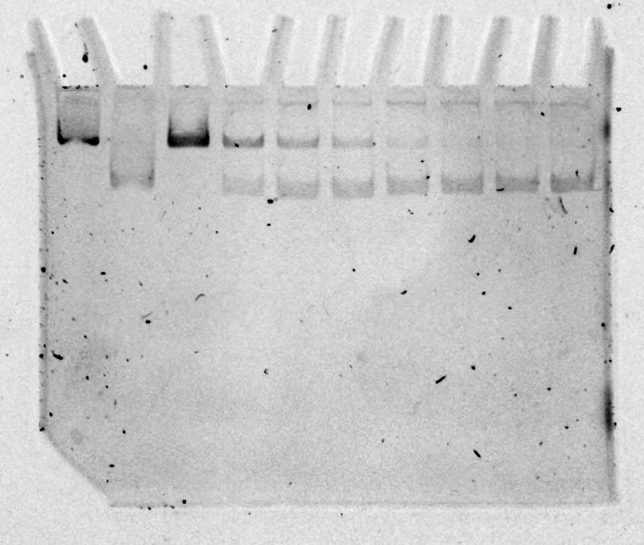
**

**Extended Data Figure 1c:**

**
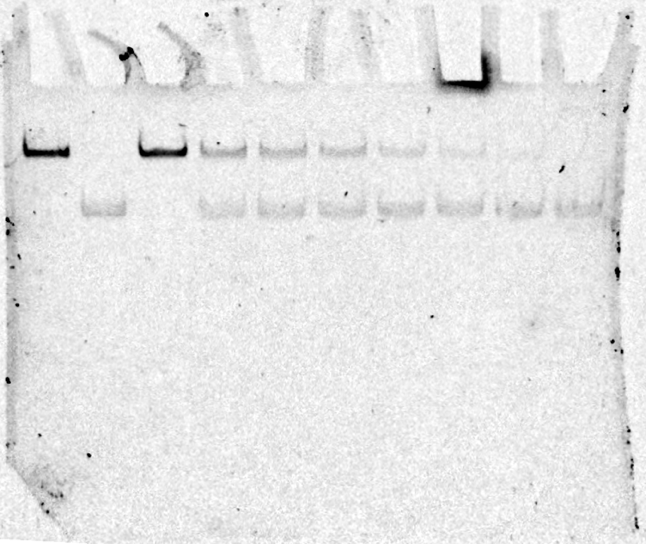
**

**Extended Data Figure 1d:**

**
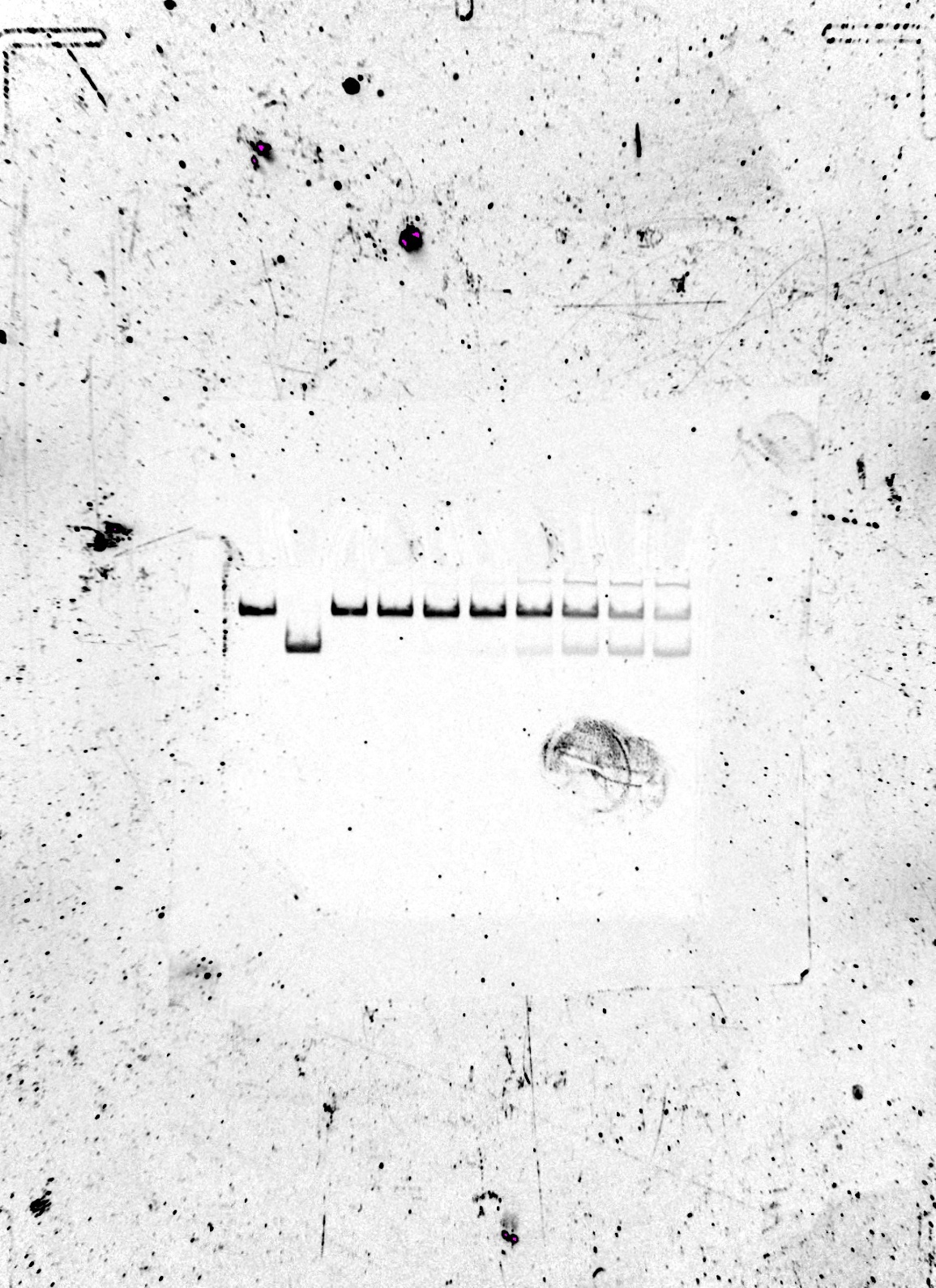
**

**Extended Data Figure 1e:**


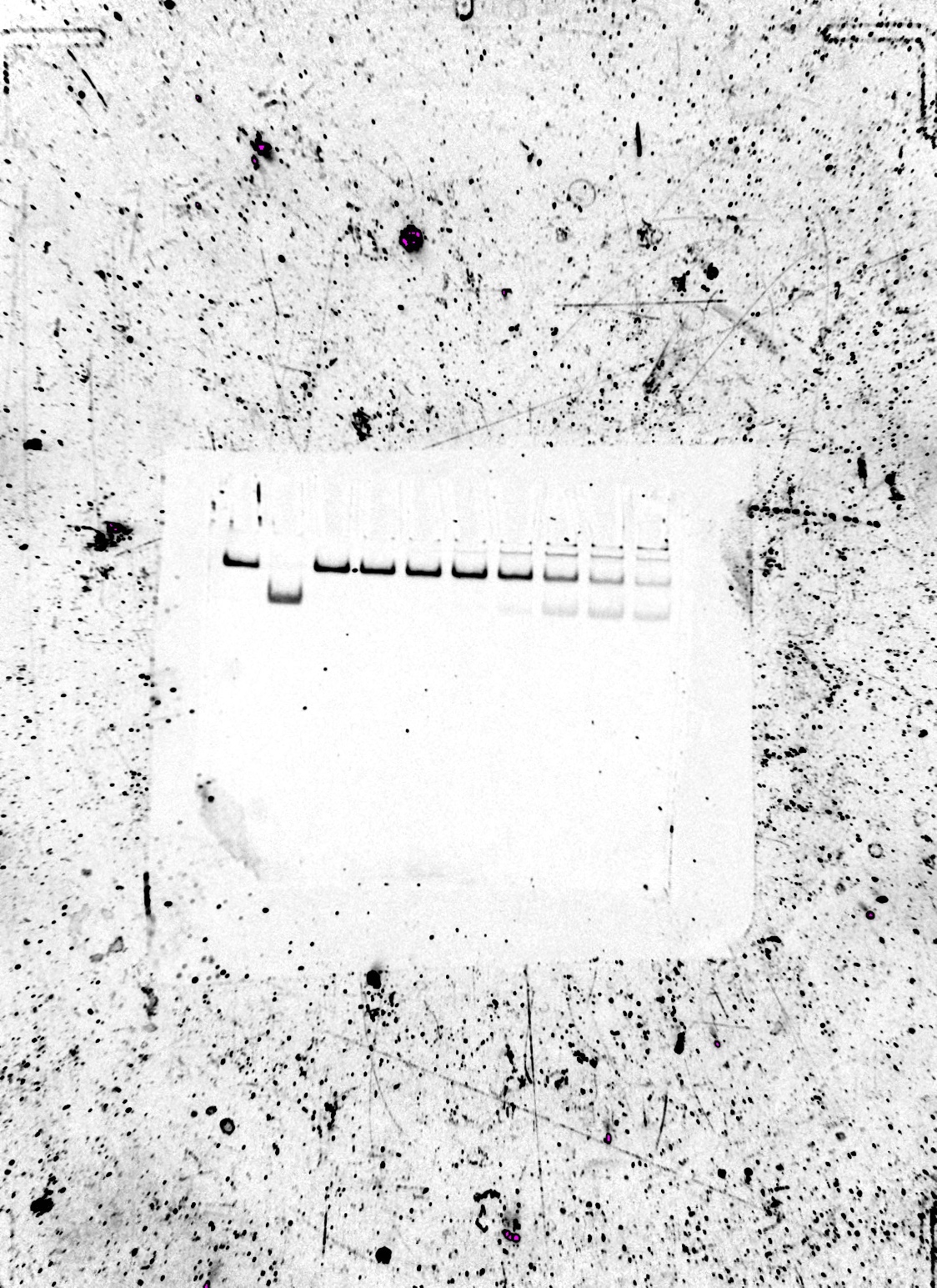


**Extended Data Figure 1f:**


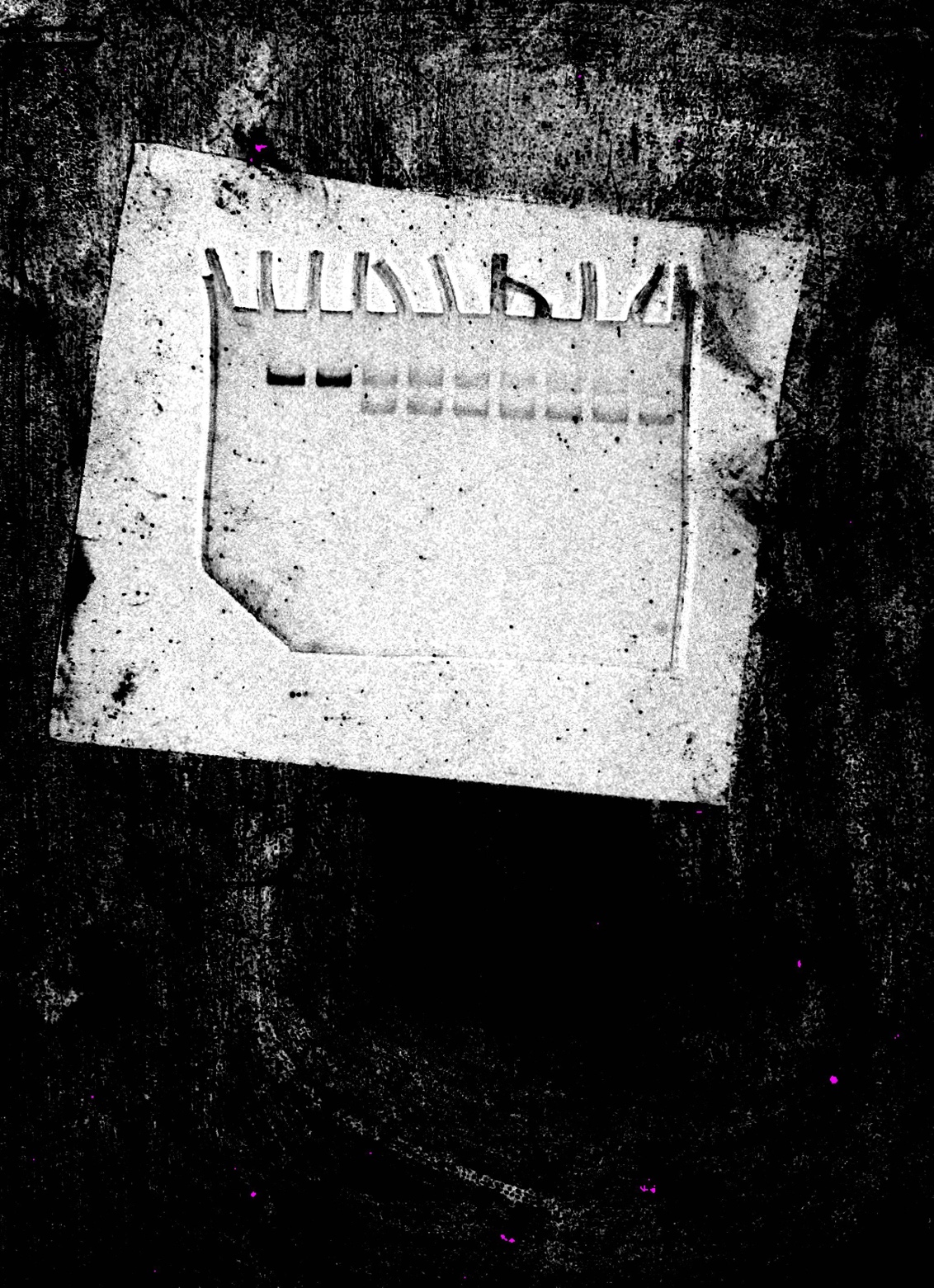


**Extended Data Figure 1h:**

**
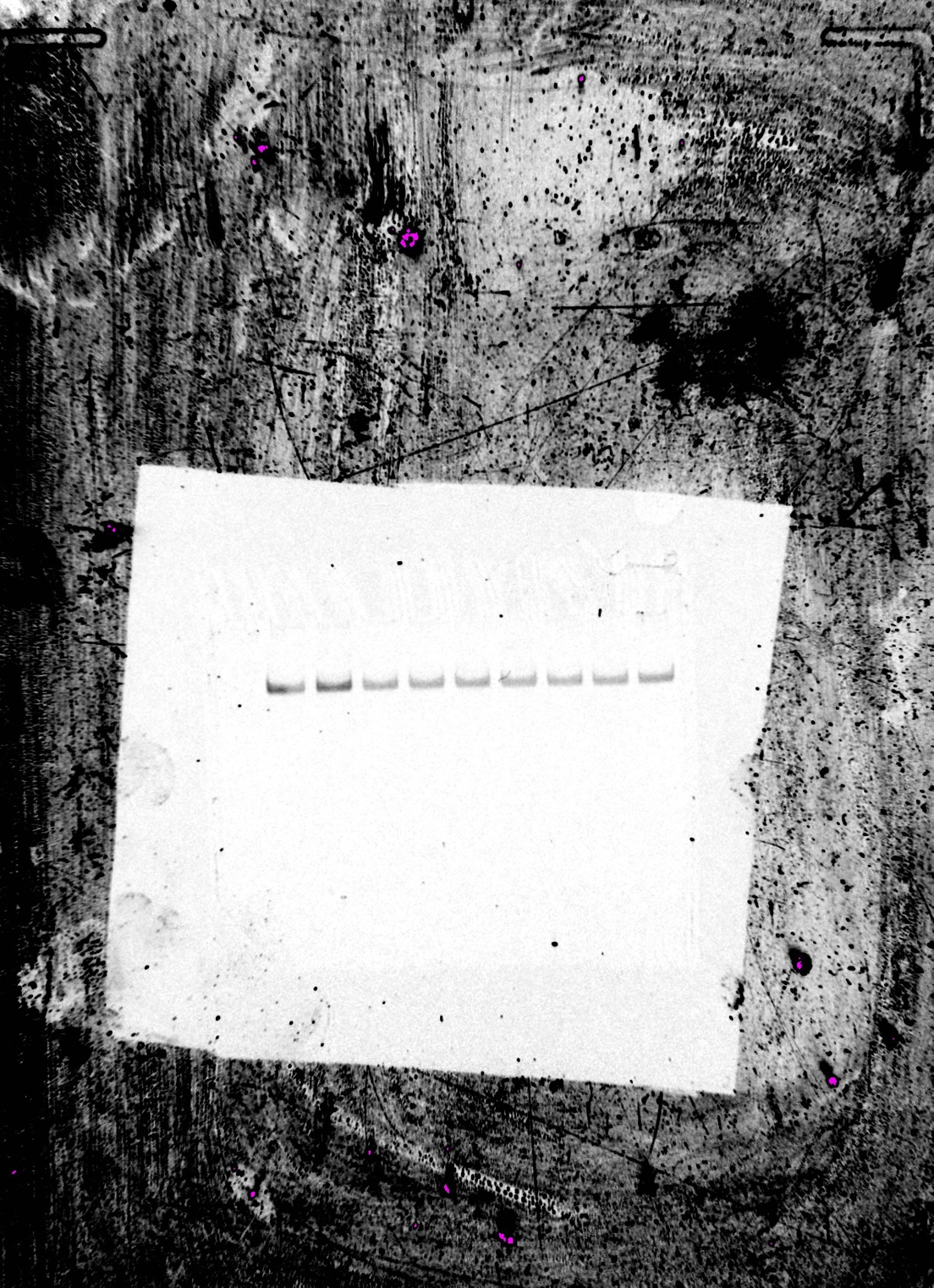
**

**Extended Data Figure 1i:**

**
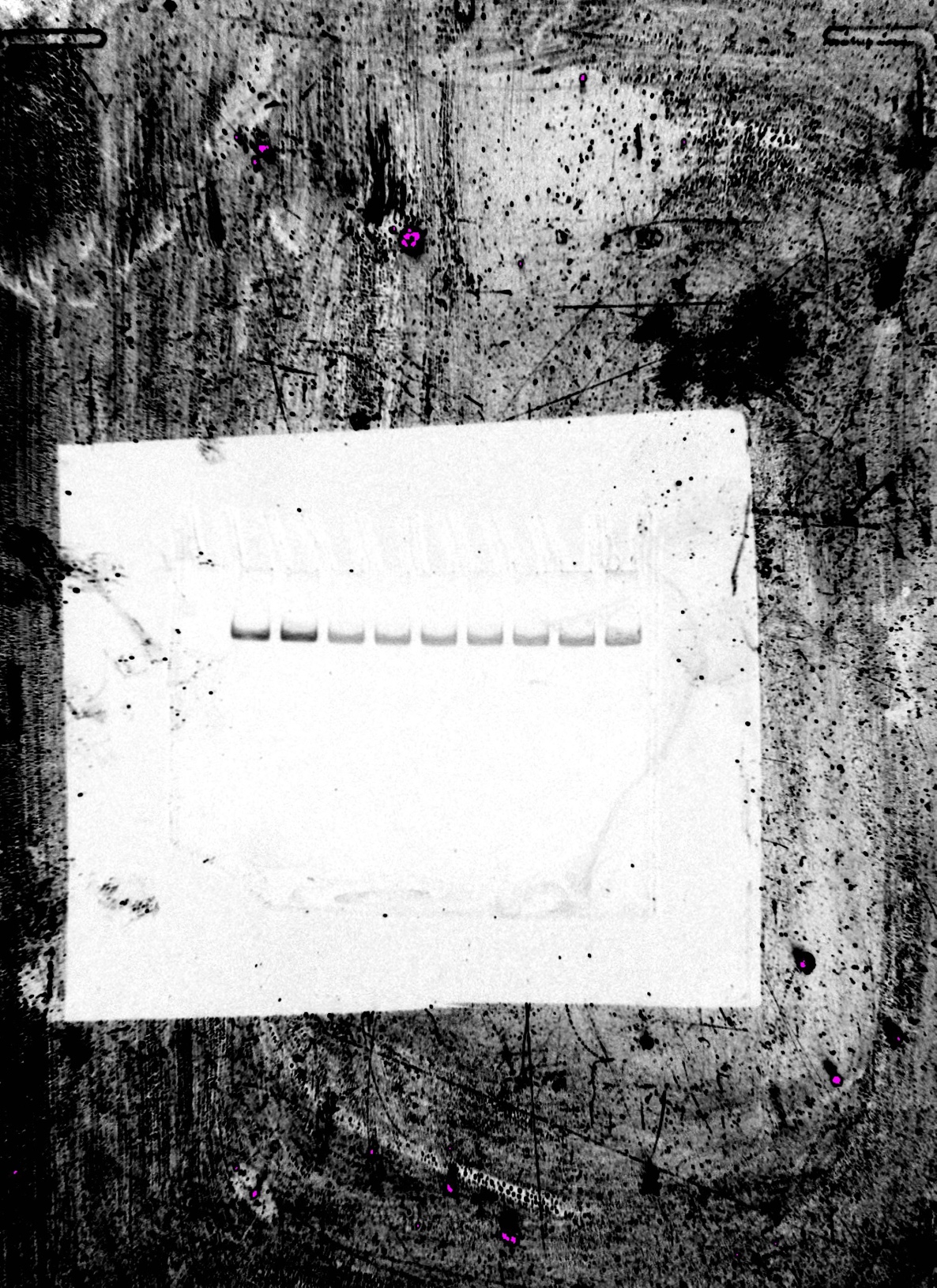
**

**Extended Data Figure 1j:**

**
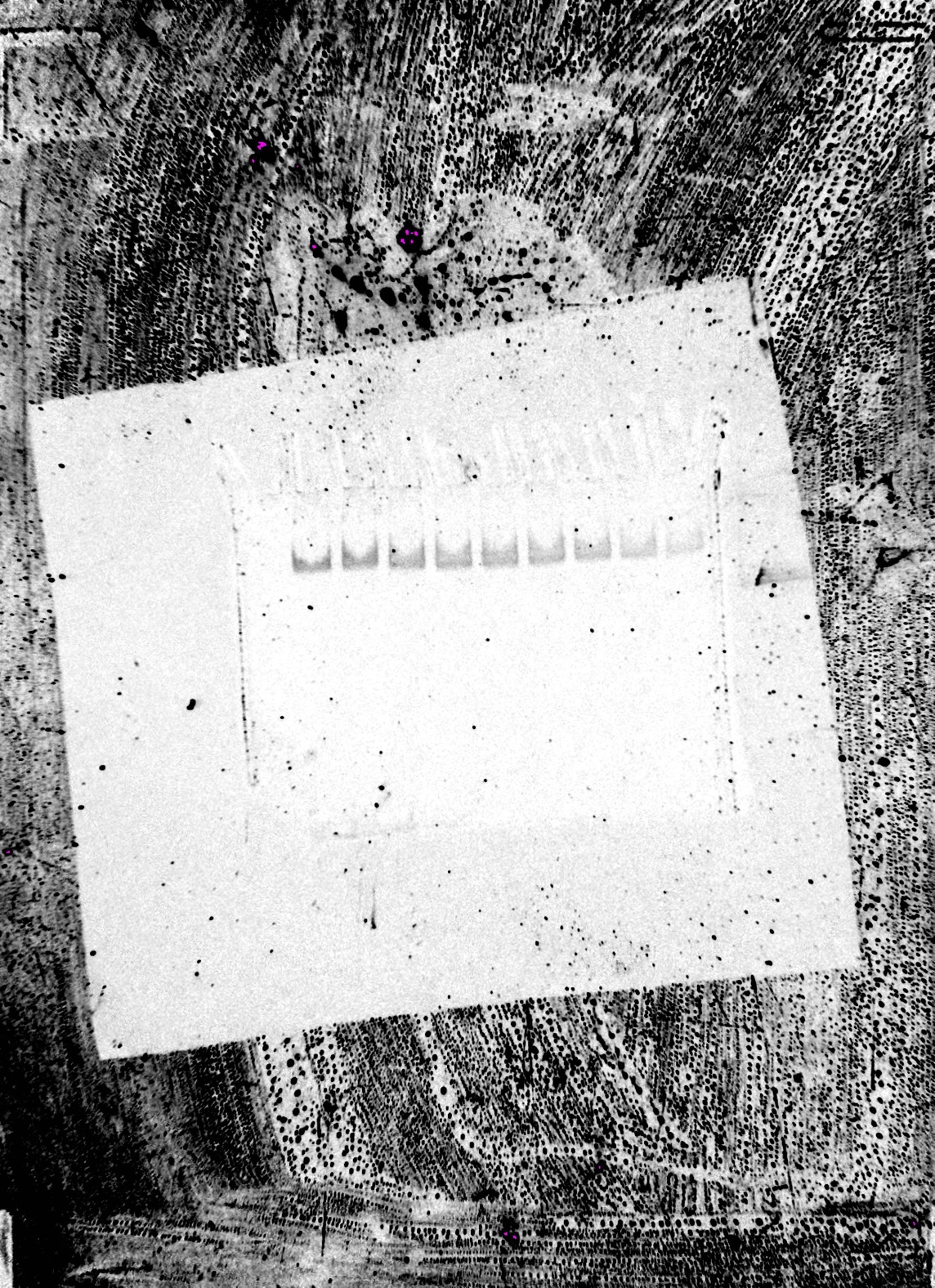
**

**Extended Data Figure 1k:**

**
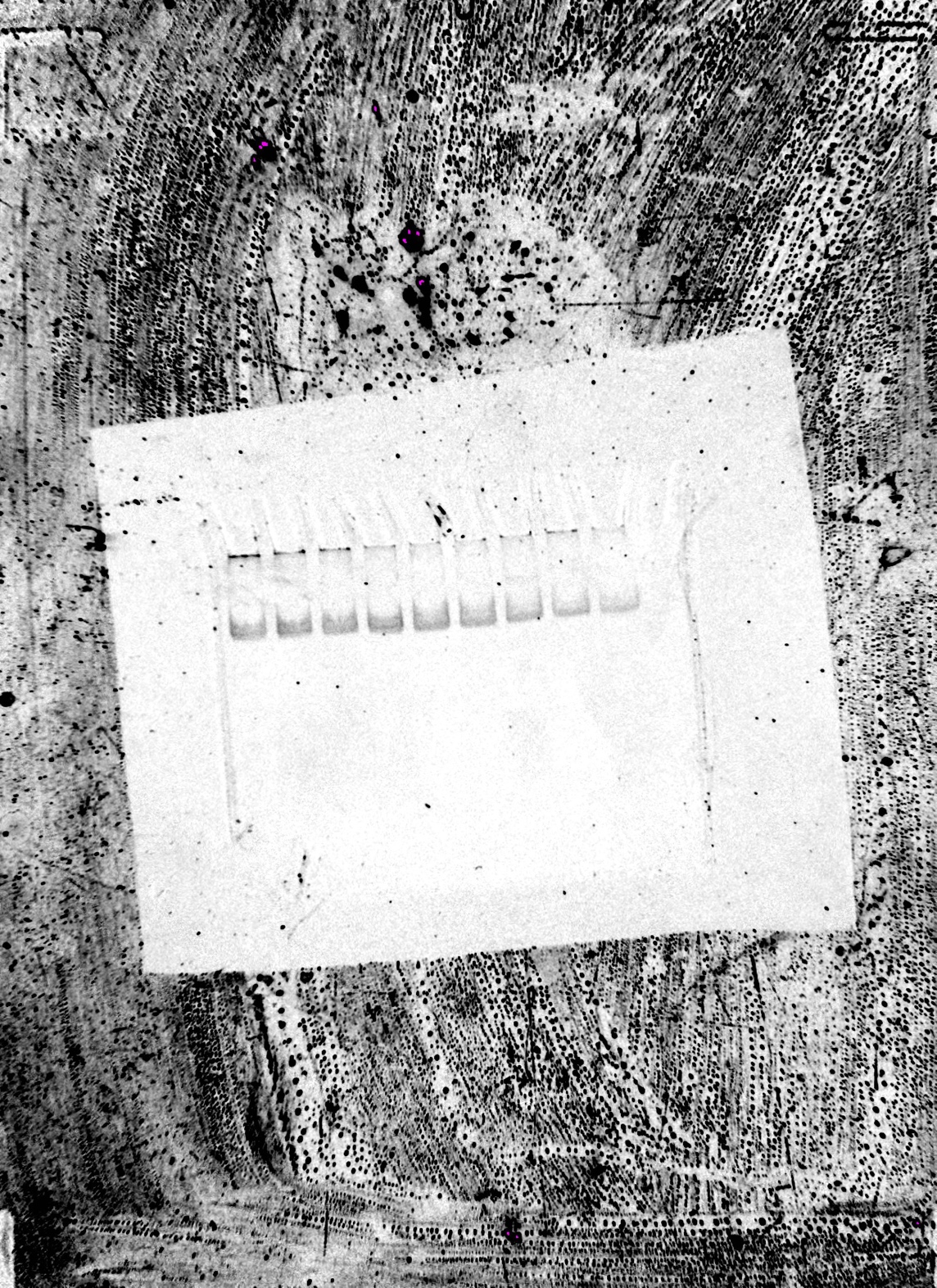
**

**Extended Data Figure 1l &m:**

**
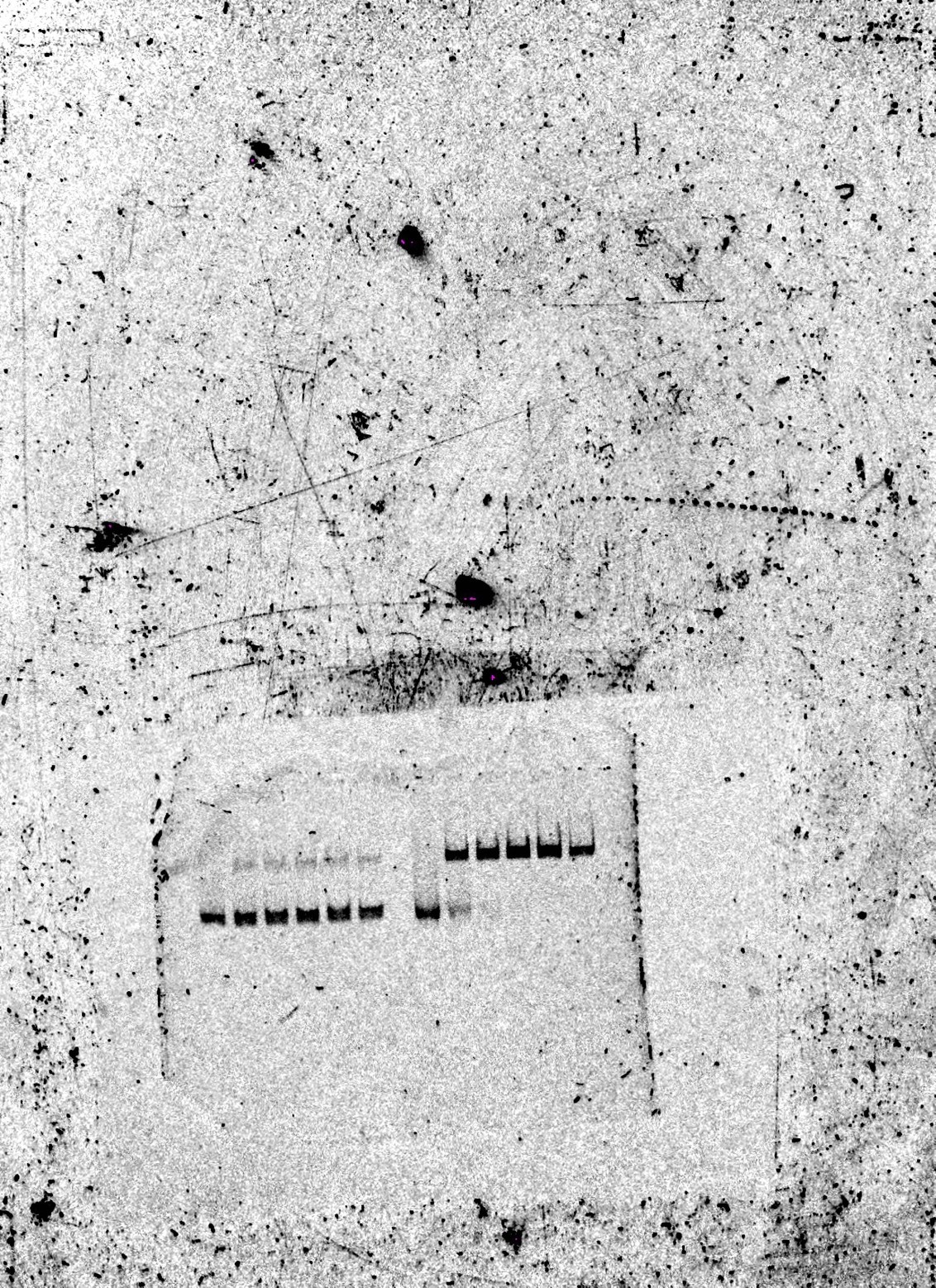
**

**Extended Data Figure 7i:**

**
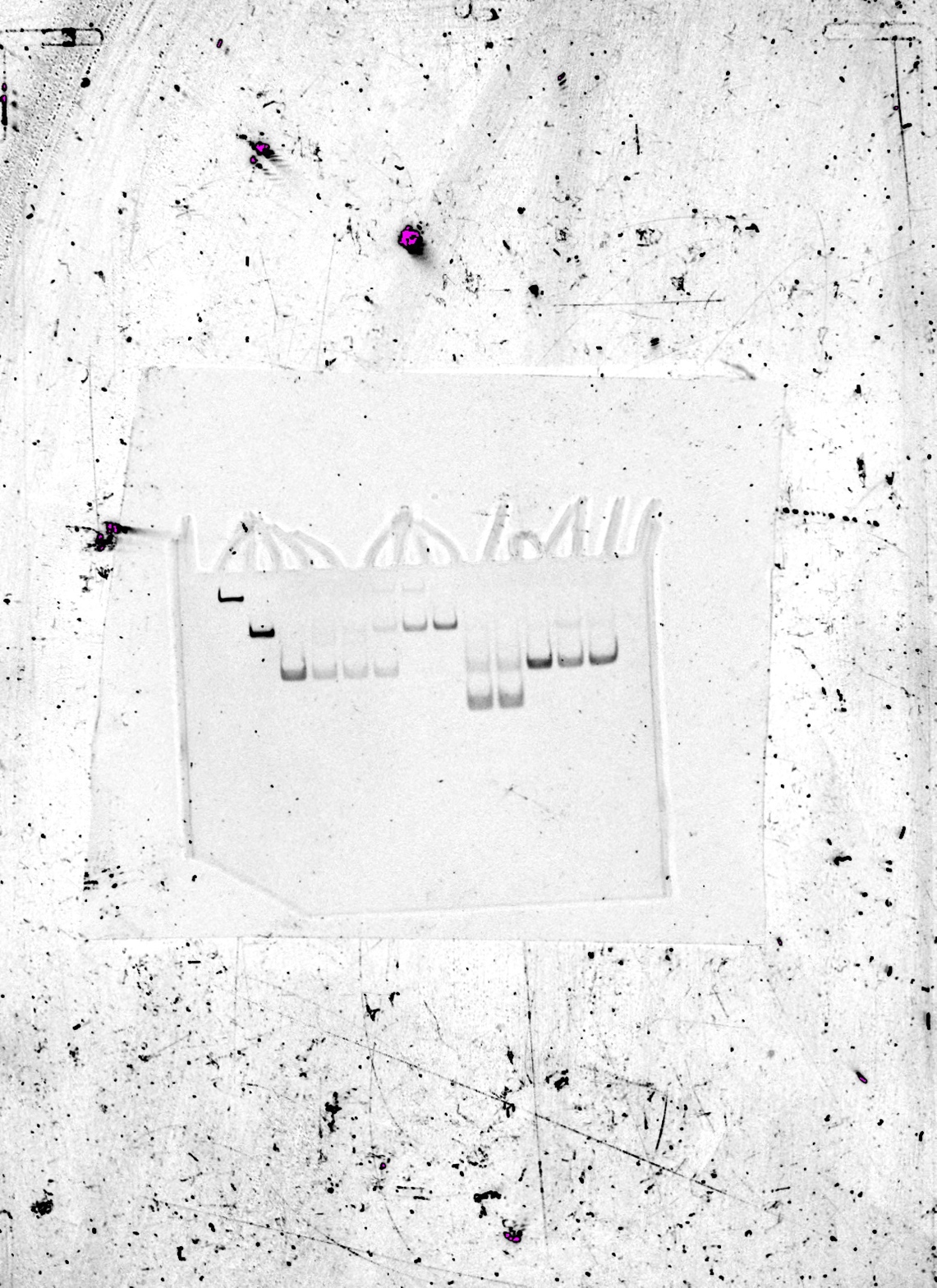
**

**Extended Data Figure 7j:**

**
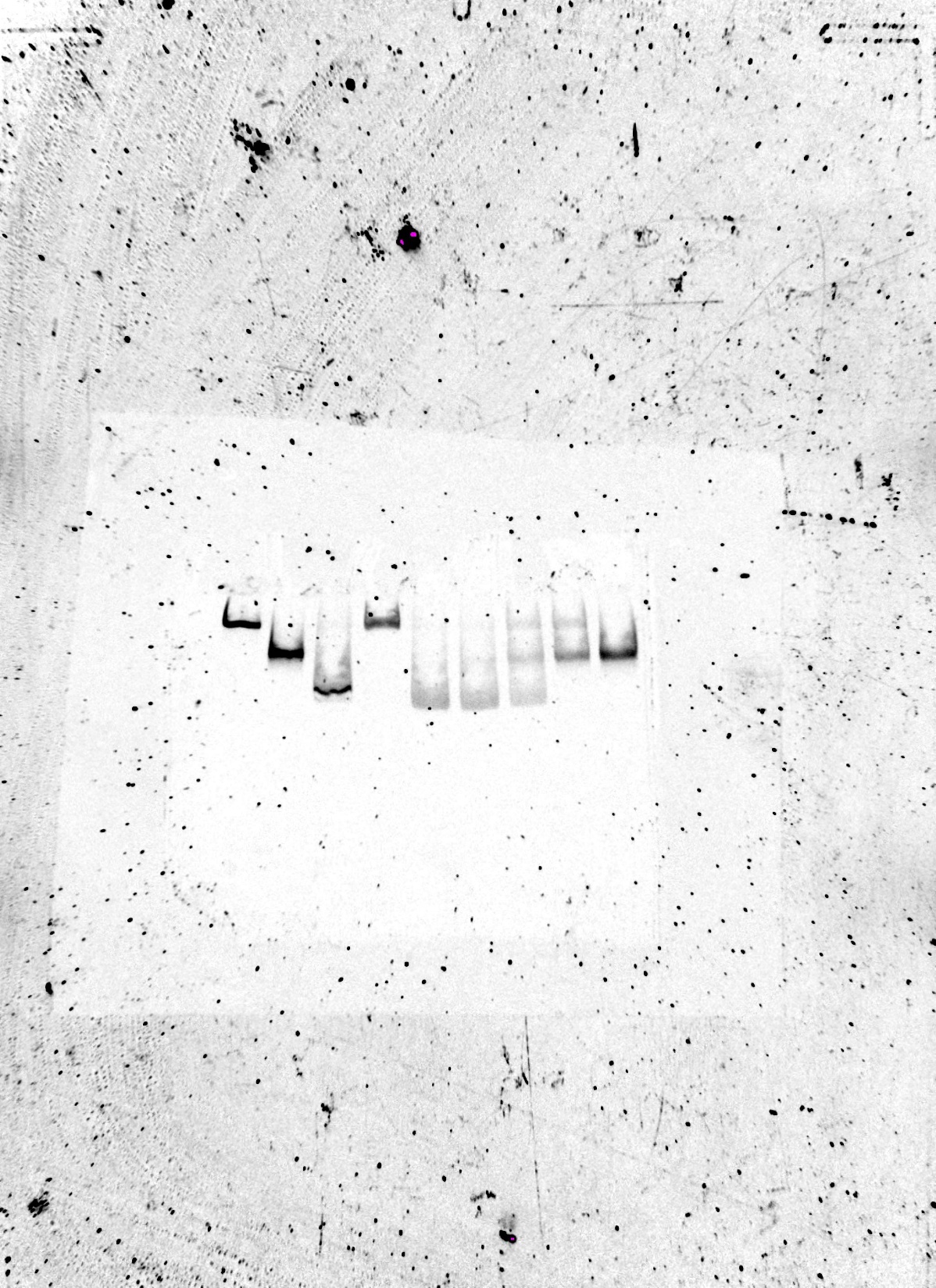
**

**Extended Data Figure 9a:**

**
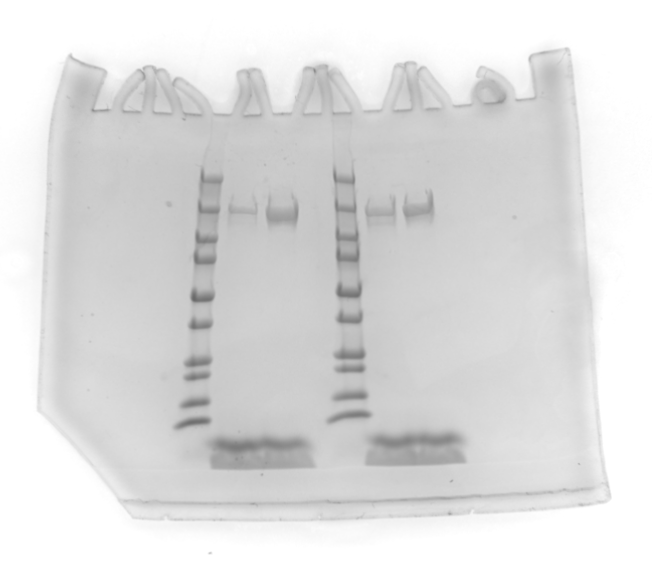
**

Coomassie scan

**
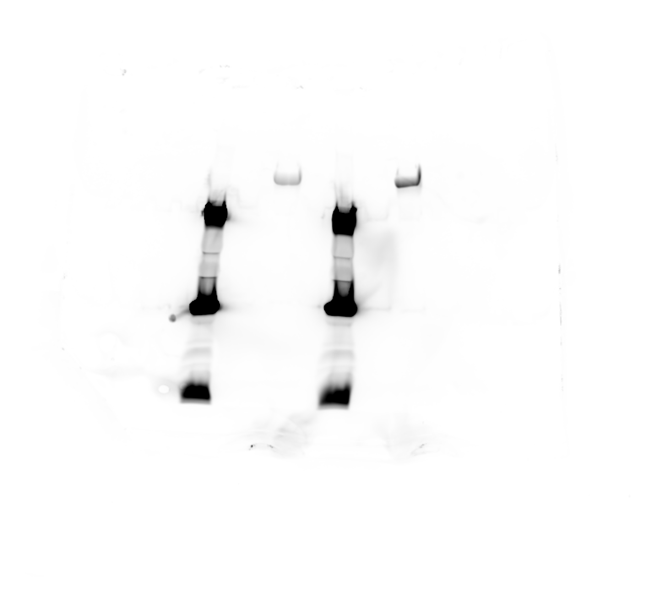
**

Cy3 scan


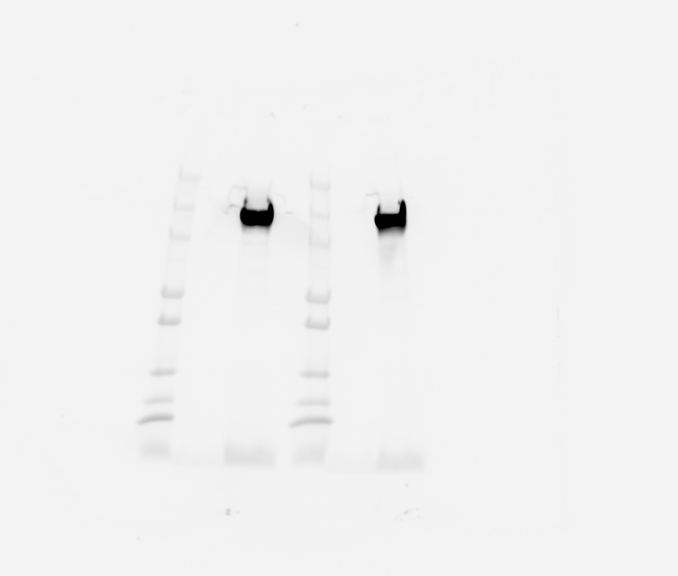


Cy5 scan

**Extended Data Figure 10e:**


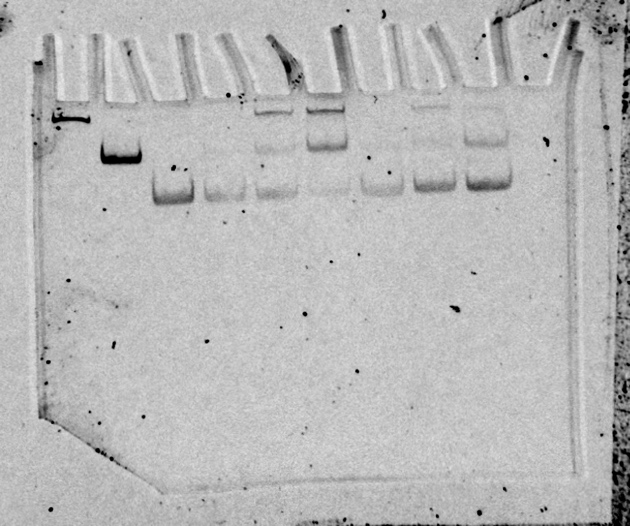


**Extended Data Figure 10f:**


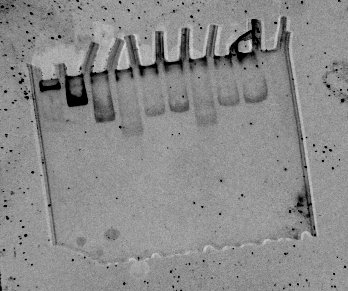


Supplementary Video 1. Video pertaining to Figure 2b-d; 360^o^ rotation of 4.4 Å electron density of (-)SC ON-dCas9 complex with Cas9 structural model. Additional rotation of 3.1 Å focus refinement map of (-)SC ON-dCas9 complex. Annotations of modelled Cas9 domains.

Supplementary Video 2. Video pertaining to Figure 2g & h; morph of structure from our study to pre-catalytic state of Cas9 (PDB = 6O0Z), highlighting 15 Å swing of HNH domain closer to target strand scissile phosphate.

Supplementary Table 1

DNA/RNA sequences used in this study for minicircle assembly, sgRNA, crRNA synthesis, tracrRNA and sgRNA *in vitro* transcription.

| **Name** | **Sequence** (5’->3’) |
| --- | --- |
| EMX1-1 ON linear DNA (Cryo-EM/AFM) | /5Phos/CCCTTCTTCT TCTGCTCGGA CTCTTTAATAGT TAATAATTTAAT AGTTTGAAATTT AATAGTTAACAA TTTAATAGTT ATTAATTTAA TAGTTTAATA TTTAATCTTA AACAACAGAT ATCATCTC |
| EMX1-1 ON splint DNA (Cryo-EM/AFM) | /5Phos/CAGAAGAAGA AGGGGAGATG ATATCTGTT |
| EMX1-1 OT1 linear DNA (Cryo-EM/AFM) (red = mismatched bases) | /5Phos/CCCTTGTTCT CTTGCTCTGA CTTTTTAATA GTTAATAATT TAATAGTTTG AAATTTAATA GTTAACAATT TAATAGTTAT TAATTTAATA GTTTAATATT TAATCTTAAA CAACAGATAT CATCTC |
| EMX1-1 OT1 splint DNA (Cryo-EM/AFM) | /5Phos/CAagAGAAcA AGGGGAGATG ATATCTGTT |
| EMX1-1 OT2 linear DNA (Cryo-EM/AFM) (red = mismatched bases) | /5Phos/CCCTTCTTCT CCTGCTCCAA CTTTTTAATA GTTAATAATT TAATAGTTTG AAATTTAATA GTTAACAATT TAATAGTTAT TAATTTAATA GTTTAATATT TAATCTTAAA CAACAGATAT CATCTC |
| EMX1-1 OT2 splint DNA (Cryo-EM/AFM) | /5Phos/CAGGAGAAGA AGGGGAGATG ATATCTGTT |
| EMX1-1 ON linear DNA (Cy3 labelled for cleavage assays) | /5Phos/GAGATGATAT CTGTTGTTTA AGATTAAATA TTAAACTATT AAATTAATAA CTATTAAATT GTTAACTATT AAATTTCAAA CTATTAAATT ATTAACTAAT AAAGAGTCCG AGCAGAAGAA GAAGGG |
| EMX1-1 ON splint DNA (Cy3 labelled for cleavage assays) | /5Phos/TATTTAATCT TAAACAACAG ATATCATCTC CCCTTCTTCT TCTGCTCGGA CTCTTTA/iAmMC6T/TA |
| EMX1-1 OT1 linear DNA (Cy3 labelled for cleavage assays) | /5Phos/GAGATGATAT CTGTTGTTTA AGATTAAATA TTAAACTATT AAATTAATAA CTATTAAATT GTTAACTATT AAATTTCAAA CTATTAAATT ATTAACTAAT AAAaAGTCaG AGCAagAGAA cAAGGG |
| EMX1-1 OT1 splint DNA (Cy3 labelled for cleavage assays) | /5Phos/TATTTAATCT TAAACAACAG ATATCATCTC CCCTTgTTCT ctTGCTCtGA CTtTTTA/iAmMC6T/TA |
| EMX1-1 OT2 linear DNA (Cy3 labelled for cleavage assays) | /5Phos/GAGATGATAT CTGTTGTTTA AGATTAAATA TTAAACTATT AAATTAATAA CTATTAAATT GTTAACTATT AAATTTCAAA CTATTAAATT ATTAACTAAT AAAAAGTTGG AGCAGGAGAA GAAGGG |
| EMX1-1 OT2 splint DNA (Cy3 labelled for cleavage assays) | /5Phos/TATTTAATCT TAAACAACAG ATATCATCTC CCCTTCTTCT CCTGCTCCAA CTTTTTA/iAmMC6T/TA |
| EMX1-1 OT3 linear DNA (Cy3 labelled for cleavage assays) | /5Phos/GAGATGATAT CTGTTGTTTA AGATTAAATA TTAAACTATT AAATTAATAA CTATTAAATT GTTAACTATT AAATTTCAAA CTATTAAATT ATTAACTAAT AAAtctcCCG gGCAGcAGAA GAAGGG |
| EMX1-1 OT3 splint DNA (Cy3 labelled for cleavage assays) | /5Phos/TATTTAATCT TAAACAACAG ATATCATCTC CCCTTCTTCT gCTGCcCGGg agaTTTA/iAmMC6T/TA |
| EMX1-1 OT4 linear DNA (Cy3 labelled for cleavage assays) | /5Phos/GAGATGATAT CTGTTGTTTA AGATTAAATA TTAAACTATT AAATTAATAA CTATTAAATT GTTAACTATT AAATTTCAAA CTATTAAATT ATTAACTAAT AAAGAGTCCG ttCAGAtGAA GAgGGG |
| EMX1-1 OT4 splint DNA (Cy3 labelled for cleavage assays) | /5Phos/TATTTAATCTT AAACAACAGA TATCATCTCC CCcTCTTCaT CTGaaCGGAC TCTTTA/iAmMC6T/TA |
| EMX1-1 sgRNA forward strand for *in vitro* transcription | ATGTAATACG ACTCACTATA GAGTCCGAGC AGAAGAAGAA GTTTTAGAGC TAGAAATAGC AAGTTAAAAT AAGGCTAGTC CGTTATCAAC TTGAAAAAGT GGCACCGAGT CGGTGCTTT |
| EMX1-1 sgRNA reverse strand for *in vitro* transcription | AAAGCACCGA CTCGGTGCCA CTTTTTCAAG TTGATAACGG ACTAGCCTTA TTTTAACTTG CTATTTCTAG CTCTAAAACT TCTTCTTCTG CTCGGACTCT ATAGTGAGTC GTATTACAT |
| Lambda 2 crRNA | /5AmMC6/GUGAUAAGUG GAAUGCCAUG guuuuagag cuaugcuguu uug |
| SpCas9 tracrRNA forward strand for *in vitro* transcription | ATGTAATACG ACTCACTATA GGACAGCATA GCAAGTTAAA ATAAGGCTAG TCCGTTATCA ACTTGAAAAA GTGGCACCGA GTCGGTGCTT TTT |
| SpCas9 tracrRNA reverse strand for *in vitro* transcription | AAAAAGCACC GACTCGGTGC CACTTTTTCA AGTTGATAAC GGACTAGCCT TATTTTAACT TGCTATGCTG TCCTATAGTG AGTCGTATTA CAT |
| Lambda DNA Endcap #1 | AGGTCGCCGC CCGGAGTTGA ACG/iBiodT//iBiodT/T/iBiodT/T/iBiodT/A CGTTCAACTC C |
| Lambda DNA Endcap #2 | GGGCGGCGAC CTCAAGTTGG ACAA/iBiodT/T/iBiodT/T/iBiodT//iBiodT/ TGTCCAACTT G |
